# Cyanide intoxication as part of smoke inhalation - a review on diagnosis and treatment from the emergency perspective

**DOI:** 10.1186/1757-7241-19-14

**Published:** 2011-03-03

**Authors:** Pia Lawson-Smith, Erik C Jansen, Ole Hyldegaard

**Affiliations:** 1 Laboratory of Hyperbaric Medicine, Department of Anesthesia, Center of Head and Orthopaedics, University Hospital Rigshospitalet, Blegdamsvej, Copenhagen, 2100, Denmark; 2 Hyperbaric Unit, Department of Anesthesia, Center of Head and Orthopaedics, University Hospital Rigshospitalet, Copenhagen, 2100 Denmark

## Abstract

This paper reviews the current literature on smoke inhalation injuries with special attention to the effects of hydrogen cyanide. It is assumed that cyanide poisoning is still an overlooked diagnosis in fire victims. Treatment against cyanide poisoning in the emergency setting should be given based on the clinical diagnosis only. Oxygen in combination with a recommended antidote should be given immediately, the first to reduce cellular hypoxia and the second to eliminate cyanide. A specific antidote is hydroxycobalamin, which can be given iv. and has few side effects.

## The most common occurrence of cyanide poisoning

Several reports have shown that persons admitted to hospital due to fire accidents may have been exposed to carbon monoxide (CO) as well as cyanide (CN) [1-3]. In fact, it has been reported that the most common source of CN poisoning in humans arise from exposure to fires [4]. In fires CN is developed when the temperature reaches 315°C (600°F) and is released from the toxic fumes in the gaseous form, i.e. hydrogen cyanide (HCN) which may then be inhaled by the victim [1]. HCN is developed from an incomplete combustion of any material containing nitrogen [5] such as plastic, vinyl, wool or silk [6]. It is worth noticing that when cotton burns it develops 130 μg HCN/g, paper 1100 μg HCN/g and wool 6300 μg HCN/g. One has to be aware that HCN is still produced when the fire is only glowing embers [7].

## Symptoms of cyanide poisoning

HCN is easily absorbed from all routes of exposure [8]. Since CN is a small lipid soluble molecule and mainly undissociated, distribution and penetration of CN into cells is rapid. CN can be distributed in the body within seconds and death can occur within seconds or minutes after a large dose [9,10]. Initially, the symptoms include a brief period of hyperpnoea, due to direct stimulation of the chemo receptors of the carotid and aortic bodies by CN [11]. CN also stimulates the nociceptors, leading to a brief sensation of dryness and burning in the nose and throat [12]. In milder cases of CN poisoning the symptoms are headache, nausea, vertigo, anxiety, altered mental status, tachypnea, hypertension and there may be an odour of bitter almonds in the patients expiration. In more severe cases the patient will have dyspnoea, bradycardia, hypotension and arrhythmia. In most severe cases the patients symptoms are unconsciousness, convulsions, cardiovascular collapse followed by shock, pulmonary oedema and death [6]. Death is due to respiratory arrest but the heart invariably outlasts respiration and may continue to beat for as long as 3-4 min. after the last gasp [8,12].

Virtually all patients with severe, acute CN poisoning die immediately. Autopsy findings include petechial, subarachnoid or subdural haemorrhages [13]. As very few people survive severe CN poisoning, reports of late neurological sequelae are rare.

CN poisoning in mild degrees is recognized as a cause of permanent neurological disability, ranging from various extrapyramidal syndromes to post-anoxic vegetative states [14]. Most cases develop over many years. Both parkinsonian symptoms and a dystonia syndrome have been observed [15-18].

## Mechanism of toxicity of CN

The similarity between CO and CN is the ability to bind iron ions. However, where CO impairs the ability of erythrocytes to transfer oxygen, CN binds to erythrocytes but does not affect the oxygen transfer. Both CO and CN affect the mitochondria by binding to the enzyme cytochrome-*c *oxidase a, a3 (CCO), the terminal enzyme complex of the respiratory chain in complex IV [10]. The active (O_2_-binding) site of CCO is binuclear, consisting of heme *a*_3 _and Cu_B _[19]. CO binds to the reduced form of CCO and CN binds to either the reduced CCO heme (Fe^2+^) or oxidized heme (Cu_B_^2+^) [20-22]. The primary effect of CN is a blocking of the mitochondrial respiration chain and the formation of intracellular adenosine triphosphate (ATP) [10]. The result is cytotoxic hypoxia caused by the inhibition of CCO by the high affinity of CN to heme *a*_*3 *_of the enzyme. The effect is a structural change and a reduced activity of the enzyme and an increase in lactate production resulting in metabolic acidosis [23,24].

## Diagnosis

CN poisoning seems to be an overlooked diagnosis in fire victims. In 1991, Baud showed that persons from fire accidents were poisoned by both CN and CO [25]. The diagnosis of CN poisoning presents a dilemma for first-response emergency personnel. Clinicians are often able to diagnose CO poisoning by either arterial- or venous blood sampling measuring carboxyhaemoglobin or by oximetry although the latter may be unreliable [26]. Diagnosing CN poisoning however, remains a challenge in the emergency setting. At the same time immediate treatment is of outmost importance. Given the fact that methods to detect and measure CN in blood are usually not readily available and that patients may often be exposed to both CO and CN, clinicians have to rely on the presenting symptoms and the general clinical status of the patient. In patients hospitalised with a history of fire accident, combined with severe neurological symptoms such as reduced Glasgow Coma Scale (GCS) Scoring and either soot particles in the mouth or tracheal expectoration, is likely to be an indicator of concomitant CN poisoning [23]. Baud et al. found that the concentration of lactate increases proportionally with the amount of CN poisoning because of the metabolic acidosis [27].

Based on these observations and given the fact that whole blood CN measurements may not be available, the patient admitted to hospital after exposure to fire combined with smoke inhalation injuries, supplementary CN intoxication should be suspected if two or more of the following criteria are fulfilled:

1) Signs of neurological incapacitation such as altered mental status, unconsciousness and convulsions

2) Soot in the mouth or expectoration

3) Fire accident patents where arterial blood sampling reveal metabolic acidosis with a lactate above 8 mmol/l as the concentration of lactate increases proportional with the rate of CN poisoning. A lactate of 10 mmol/l is a sensitive and specific indicator of CN intoxication [23].

Currently, two methods of whole blood CN analysis dominates the literature:

One method is the Conway/microdiffusion method where test material is whole blood. CN is liberated from the blood into the gas phase and subsequently bound to hydroxycobalamin (OHCob) forming cyanocobalamin (CNCob). The concentration of CNCob can be read my means of a spectrophotometer [28]. Results are available within a 2-h period.

The other method is isotope-dilution gas chromatography-mass spectrometry (ID GC/MS) that is an automated procedure where test material is whole blood. Samples are prepared and analysed within a 2-h period [29].

With the current available methods for the analysis of CN blood concentrations, one may conclude that in the clinical setting it takes hours before a result may be available for the treating doctor [30]. Furthermore, CN is an unstable molecule and has an elimination half-life of 1 hour in blood in vivo. Therefore determination of CN in blood requires rapid sampling and analysis [25,27].

## Treatment

The treatment of CN poisoning is aiming at basic life support including 100% oxygen, assisted ventilation if the patient is unconscious (GCS < 8) or the airway seems compromised, decontamination, correction of acidosis and blood pressure support [31,32] combined with the use of an antidote. Currently there are four types of antidotes. These include OHCob, sodium thiosulfate, dicobalt edetate and methaemoglobin forming antidotes. Initial evaluation of antidotal efficacy is based on correction of hypotension and lactic acidosis and the final outcome rests on the degree of permanent central nervous system injury [33]. The different antidotes shall be described briefly here below.

OHCob has a rapid onset of action as it dissolves into the different tissue compartments almost immediately when administered by infusion [34]. It has the advantage of not interfering with tissue oxygenation [35] and in both human and animal studies it has been shown to improve hemodynamic stability [34,36-38]. OHCob acts by covalent binding to CN and forms cyanocobalamin (CNCob) which is B12 vitamin [39,40]. CNCob is excreted through the kidneys [41]. Given iv. OHCob distributes to the erythrocytes and plasma cells and after 30 minutes it reaches the cerebrospinal fluid [42]. Side effects are red colouring of skin and urine, urticarial eczema and seldom anaphylactic chock [32]. In a series of normal human volunteers given 5 g of OHCob iv. during 20 minutes, a mild, transient, self-limiting hypertension accompanied by reflex bradycardia has been reported [38]. OHCob must not delay any other basic life support such as securing of the airways, cardiovascular support or oxygen supply [31,32]. OHCob in blood interferes with CO-oximetry measurements of COHb, methemoglobin (MetHb), and Hb-O_2_. This must be considered during OHCob treatment, particularly in smoke inhalation victims with concurrent CO exposure, because it may lead to potentially erroneous reported COHb levels. OHCob will cause an increase in measured COHb percentage values [43].

Sodium thiosulfate removes CN from the blood through the action of rhodanese [44]. Rhodanese is an enzyme located in the mitochondria mainly in the liver, kidney and skeletal muscles [45,46]. It adds a sulphur atom to CN and forms thiocyanate which is less toxic and excreted through the kidneys [47,48]. Sodium thiosulfate has limited distribution into the brain as well as limited penetration into the mitochondria, where the endogenous rhodanese is located [40,49]; accordingly sodium thiosulfate exerts its main effect in blood and plasma [50]. Sodium thiosulfate has a slow onset of action [6]. Less significant side effects such as nausea, vomiting, and injection site pain, irritation, and a burning sensation has been reported [39,51]. There is limited information available about the efficacy of sodium thiosulfate for treatment of CN poisoning [35]. No clinical trials of sodium thiosulfate are available, and efficacy has been extrapolated from case studies and series of acute CN poisoning.

Dicobalt EDTA is an efficient antidote with a high affinity to CN but it has restricted use. The mechanism of action is chelation of CN to form the much less toxic cobalt cyanide. Dicobalt EDTA has deleterious cardio-vascular side effects and is often poorly tolerated. To mitigate these side effects intravenous glucose should be co administered during treatment. The side effects are enhanced if the patient is not CN poisoned so it should be used only in very severe cases where the diagnosis is certain [32,35,40].

Amyl nitrite and sodium nitrite are methemoglobin forming antidotes, which are relatively contraindicated in smoke inhalation. Nitrite reduces blood CN by forming methemoglobin, to which CN binds with higher affinity than it does to CCO. Significant side effects such as vasodilatation and hypotension are seen during treatment. Induction of methemoglobin forming antidote treatment has the potential to impair the oxygen carrying capacity of haemoglobin [6]. In the smoke inhalation victim, with concomitant COHgb increase and possible pulmonary injury, there is an obvious added risk associated with methemoglobin formation [6].

## Adjunctive treatment of CN intoxication

Hyperbaric oxygen therapy (HBO) is recommended by UHMS as an adjunct to the treatment of CO poisoning complicated by CN poisoning [52]. HBO has been shown to improve survival and improve tissue oxygenation in the clinical as well as in the experimental settings [53] and HBO is recommended especially when supportive measures and other CN antidotes fail [54-56]. Several studies have demonstrated a protective effect of HBO therapy in experimental ischemic brain injury, and many physiological and molecular mechanisms of HBO therapy-related neuroprotection have been identified [57]. Also HBO has been shown to reduce the risk of cognitive sequelae after acute CO poisoning when HBO is given within a 24-hour period [58]. Furthermore it has been shown that HBO increases the flexibility of red blood cells (thereby improving microcirculatory perfusion), reduces tissue oedema and preserves intracellular ATP [59-62]. The binding of CN to CCO is most often referred to as being irreversible [23,32]. However, recent evidence suggests that CN binding to CCO is reversible. Where CN binding to CCO appears to be independent of the oxygen tension, there seems to be a competition between CN and nitric oxide (^•^NO). High concentrations of ^•^NO have been found to attenuate the inhibition of CCO induced by CN and CO [63,64]. In keeping with this, HBO therapy, but not normobaric oxygen, has been shown to increase the bioavailability of ^•^NO [65-69] which may show to be beneficial during CN poisoning. Whether HBO therapy holds any place in the treatment of acute CN poisoning when readily available is a matter of continued debate. In keeping with the above and the fact that patients from fires are both CO and CN poisoned we recommend HBO as well where safely available.

## Conclusion

Treatment of suspected CN poisoning presents a dilemma for medical first-response emergency personnel, as clinicians are often unable to diagnose CN poisoning in the emergency setting. Immediate treatment is of outmost importance. In summary immediate treatment includes 100% oxygen, assisted ventilation if the patient is unconscious (GCS < 8) or the airway seems compromised, decontamination, correction of acidosis and blood pressure support [31,32]. Antidotes include OHCob, sodium thiosulfate, di-cobalt EDTA and methaemoglobin-inducers. Currently, there is no international agreement of which antidote is the preferred to use but OHCob and sodium thiosulfate seem to be among the most widely accepted antidotes. OHCob is an attractive antidote due to its rapid CN binding and its lack of serious side effects, even in the absence of CN intoxication. Accordingly this is the recommended antidote treatment in Denmark to known or suspected CN poisoning. In France OHCob is given prehospital by EMS personnel but not in Denmark, as the Health Ministry has not approved it for this use [70].

## Competing interests

The authors declare that they have no competing interests.

## Authors' contributions

PL-S drafted the manuscript. All authors read and approved the final manuscript.
